# Electrical impedance tomography as a bedside assessment tool for COPD treatment during hospitalization

**DOI:** 10.3389/fphys.2024.1352391

**Published:** 2024-03-18

**Authors:** Lin Yang, Zhijun Gao, Xinsheng Cao, Shuying Sun, Chunchen Wang, Hang Wang, Jing Dai, Yang Liu, Yilong Qin, Meng Dai, Wei Guo, Binghua Zhang, Ke Zhao, Zhanqi Zhao

**Affiliations:** ^1^ Department of Aerospace Medicine, Air Force Medical University, Xi’an, China; ^2^ Department of Pulmonary and Critical Care Medicine, 986th Hospital of Air Force, Air Force Medical University, Xi’an, China; ^3^ Department of Biomedical Engineering, Air Force Medical University, Xi’an, China; ^4^ School of Biomedical Engineering, Guangzhou Medical University, Guangzhou, China; ^5^ Department of Critical Care Medicine, Peking Union Medical College Hospital, Chinese Academy of Medical Sciences, Beijing, China

**Keywords:** acute exacerbation of chronic obstructive pulmonary disease, treatment efficacy, bedside assessment, lung function, regional ventilation distribution

## Abstract

For patients with chronic obstructive pulmonary disease (COPD), the assessment of the treatment efficacy during hospitalization is of importance to the optimization of clinical treatments. Conventional spirometry might not be sensitive enough to capture the regional lung function development. The study aimed to evaluate the feasibility of using electrical impedance tomography (EIT) as an objective bedside evaluation tool for the treatment of acute exacerbation of COPD (AECOPD). Consecutive patients who required hospitalization due to AECOPD were included prospectively. EIT measurements were conducted at the time of admission and before the discharge simultaneously when a forced vital capacity maneuver was conducted. EIT-based heterogeneity measures of regional lung function were calculated based on the impedance changes over time. Surveys for attending doctors and patients were designed to evaluate the ease of use, feasibility, and overall satisfaction level to understand the acceptability of EIT measurements. Patient-reported outcome assessments were conducted. User’s acceptance of EIT technology was investigated with a five-dimension survey. A total of 32 patients were included, and 8 patients were excluded due to the FVC maneuver not meeting the ATS criteria. Spirometry-based lung function was improved during hospitalization but not significantly different (FEV1 %pred.: 35.8% ± 6.7% vs. 45.3% ± 8.8% at admission vs. discharge; *p* = 0.11. FVC %pred.: 67.8% ± 0.4% vs. 82.6% ± 5.0%; *p* = 0.15. FEV1/FVC: 0.41 ± 0.09 vs. 0.42 ± 0.07, *p* = 0.71). The symptoms of COPD were significantly improved, but the correlations between the improvement of symptoms and spirometry FEV1 and FEV1/FVC were low (R = 0.1 and −0.01, respectively). The differences in blood gasses and blood tests were insignificant. All but one EIT-based regional lung function parameter were significantly improved after hospitalization. The results highly correlated with the patient-reported outcome assessment (R > 0.6, *p* < 0.001). The overall acceptability score of EIT measurement for both attending physicians and patients was high (4.1 ± 0.8 for physicians, 4.5 ± 0.5 for patients out of 5). These results demonstrated that it was feasible and acceptable to use EIT as an objective bedside evaluation tool for COPD treatment efficacy.

## Introduction

Chronic obstructive pulmonary disease (COPD) is a severe pulmonary disease characterized by progressive airflow obstruction. It has emerged as the third leading cause of death globally (now the fourth following the COVID-19 pandemic). This respiratory disorder affects 9%–19% of adults, with many cases going undiagnosed (Varmaghani et al., 2019). A recent study revealed that approximately 99.9 million individuals in China have COPD (Wang et al., 2018). The increasing prevalence of COPD is attributed to factors such as an aging population and escalating environmental pollution, leading to a growing global disease burden. By 2030, the World Health Organization predicts that COPD will move up to become the third leading cause of death around the world, following ischemic heart disease and stroke (WHO World Health Organization, 2020). Acute exacerbations of COPD (AECOPD) are often triggered by infectious or environmental factors, necessitating hospitalization, thereby adding pressure on both patients and the social healthcare system. The treatment focus for AECOPD is symptom relief during acute exacerbations and their prevention. Common medications include bronchodilators, corticosteroids, and antibiotics. Inhalation therapy with short-acting beta-agonists and anticholinergics is typical in uncomplicated cases. Elevated blood eosinophil levels may lead to inhaled corticosteroid addition. Severe cases benefit from oral corticosteroids, improving lung function and recovery time. Antibiotic use depends on respiratory distress severity, sputum characteristics, and ventilation needs. Oxygen therapy and mechanical ventilation support are crucial adjunctive measures.

COPD is a complex and heterogeneous disease characterized by various phenotypes, including chronic bronchitis and emphysema, leading to diverse responses to treatments and disease progression (Agustí et al., 2022). According to the GOLD guidelines, lung function evaluation using a spirometry test [such as forced expiratory volume in one second (FEV1) and forced vital capacity (FVC)] is the gold standard for diagnosing COPD and monitoring disease progression (Global Initiative for Chronic Obstructive Lung Disease, 2023). Although spirometry is reproducible, it cannot differentiate between COPD phenotypes or evaluate respiratory symptoms. Questionnaire-based patient-reported outcome (PRO) assessments, like the COPD assessment test (CAT) and Quality of Life Disease Impact Scale, are often used to determine changes in the overall COPD disease status and health-related quality of life. These assessments are subjective, making it challenging to compare results across individuals. In addition, the age of patients might influence their comprehension of the questionnaires.

To assess treatment efficacy, imaging techniques could be a viable option. However, x-ray or computed tomography scans cannot be applied frequently due to radiation exposure. Electrical impedance tomography (EIT) is a novel medical imaging technique that evaluates regional lung ventilation and perfusion (Frerichs et al., 2017). By introducing imperceptible alternating currents through electrodes on the chest wall surface, EIT measures the electrical potentials and reconstructs the impedance images. This technique is based on changes in the regional air content and regional blood flow alternating the electrical impedance of lung tissue (de Castro Martins et al., 2019). So far, EIT has been used to evaluate regional ventilation in patients with various lung diseases, e.g., COPD, asthma, cystic fibrosis, and IPF (Zhao et al., 2013; Milne et al., 2017; Ngo et al., 2018; Krauss et al., 2021). Alterations in spatial and temporal ventilation distribution after bronchodilator were captured both in asthma and COPD cases (Frerichs et al., 2016; Vogt et al., 2016). Jiang et al. (2021) also used EIT to evaluate treatment efficacy on ventilation in patients with pneumonia. Up to now, no study has explored the use of EIT to assess the improvement after longer treatment for patients with COPD. The study aimed to evaluate the feasibility of using EIT as an objective bedside tool for evaluating COPD treatment effectiveness.

## Methods and materials

The prospective observational study was approved by the ethics committee of the Fourth Military Medical University (KY20224101-1). Informed consent was obtained from all subjects prior to the study. Consecutive patients admitted to the Department of Pulmonary and Critical Care Medicine of 986th Hospital of the Air Force Medical University due to AECOPD from March 2023 to October 2023 were included prospectively. The exclusion criteria included the following: difficulty in completing the lung function test, chest skin injury against the placement of the EIT electrode belt, and other contraindications to using chest EIT (e.g., cardiac pacemaker).

### Treatments and measurements

During hospitalization, hypoxemia improved with supplemental oxygen given via nasal catheter-inspired oxygen (FiO_2_ 24%–35%). Short-acting inhaled beta-2 agonists were administered, with short-acting anticholinergics salbutamol (albuterol) and ipratropium bromide. In patients with severe exacerbations, systemic corticosteroids (methylprednisolone) were used to improve lung function and oxygenation and shorten recovery time. The duration of therapy was normally no more than 5 days. Antibiotics were applied when signs of bacterial infection were presented.

Blood gases; blood tests including procalcitonin (PCT), C-reactive protein (CRP), and white blood cell (WBC); interleukin (IL)-6; lactic acid (LA); and x-ray or CT were performed at the time of admission and discharge. Modified Medical Research Council (mMRC) and COPD assessment test (CAT) scores were used to evaluate patients’ symptom status. The FVC maneuver was performed according to ATS guidelines (Graham et al., 2019).

**FIGURE 1:**
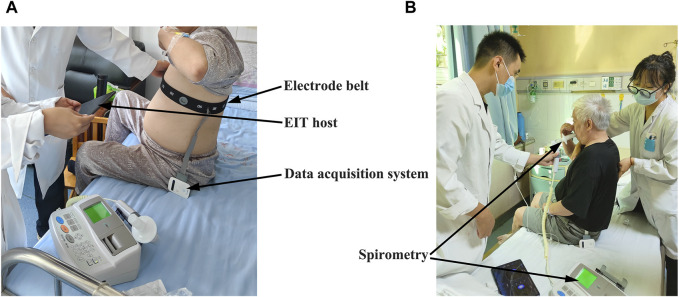
View of the EIT measurement on the patient with chronic obstructive pulmonary disease (COPD) during hospitalization: **(A)** attachment and adjustment of the EIT electrode belt to ensure good electrode–skin contact; **(B)** implement of the forced vital capacity (FVC) maneuver. The locations of the EIT acquisition system, electrode belt, and spirometer are highlighted with arrows.

EIT measurements were conducted according to the manufacturer’s manual at the time of admission and before the discharge simultaneously when the FVC maneuver was conducted. The device and measurement setup details were previously published (Yang et al., 2023a; Yang et al., 2024). In brief, a belt with 16 equidistantly fixed electrodes was placed around the chest in one transverse plane at the level of the 5th intercostal space at the parasternal line, as shown in Figure 1. Raw EIT data were acquired with VenTom-100 (MidasMED Biomedical Technology, Suzhou, China) at a scan rate of 20 images/s using excitation currents of 1 mArms applied through opposite electrodes. Image reconstruction was accomplished by the GREIT algorithm (Adler et al., 2009). The baseline for image reconstruction was obtained individually for each subject during quiet tidal breathing.

### EIT data analysis

Lung regions were identified as the pixels with a relative impedance change Δ*Z* higher than 20% of the maximum Δ*Z* (Zhao et al., 2018). The differences between the maximum relative impedance change Δ*Z* values achieved after the forced full inspiration to total lung capacity and the lowest relative Δ*Z* values during the succeeding maximum expiration to residual lung volume were calculated in the pixels of the lung regions. These values reflected the pixel values of forced expiratory vital capacity (*FVC*_*EIT*_). Δ*Z* in arbitrary unit (AU) was normalized to milliliter by comparing the sum of all pixel *FVC*_*EIT*_ values and the *FVC* measured with spirometry (Eq. 1).

$$Z\left(in\;ml\right)=Z\;\left(in\;AU\right)\times FVC\;\left(in\;ml\right)/{FVC}_{EIT}\;\left(in\;AU\right).$$ (1)

Another parameter determined from the forced full inspiration was the forced expiratory volume in 1 s (*FEV1*_*EIT*_), which was calculated as the difference between the rel. Δ*Z* values after 1 s of forced full expiration and the value at residual lung volume at the beginning of the expiration limb of the maneuver in the lung pixels. Furthermore, the global time points of 25% and 75% of FVC were identified (*MEF25* and *MEF75*). The mean flow was calculated in the pixel level and denoted as *MEF25-75*_*EIT*_. Together with pixel-wise *FEV1*_*EIT*_/*FVC*_*EIT*_, a total of four functional EIT images were calculated (fEIT FVC, fEIT FEV1, fEIT MEF25-75, and fEIT FEV1/FVC). To characterize the dispersion of the fEIT images, i.e., the heterogeneity of their spatial distribution, the global inhomogeneity (*GI*) index (Zhao et al., 2009) was calculated for each type of fEIT. For fEIT FEV1/FVC, the number of pixels with values < 0.7 was calculated in percentage to the total number of pixels in the lung regions. This parameter was denoted as Abnormal%.

The time constant map was calculated as described in a previous study (Karagiannidis et al., 2018; Strodthoff et al., 2022). In brief, for every pixel within the lung regions, the regional time constant was calculated by fitting the following exponential equation: (Eq. 2).

Zt=Z0∙e−tτ+c, (2)

where *Z*(*t*) is the relative impedance in a pixel that is within the lung at time point *t*, *Z*_0_ is the impedance at the start of expiration, *t* represents the time from the end-inspiration to the end-expiration, *τ* denotes the regional time constant, and c denotes the end-expiratory volume. Instead of calculating it for every tidal breath, as proposed earlier, the time constant map was calculated from the FVC data in the present study. The regional median *τ* (*τ*_*med*_) and interquartile range (*τ*_*iqr*_) were calculated.

To understand the acceptability of EIT measurements, two surveys for attending doctors and patients were designed including the ease of use, feasibility, and overall satisfaction level. It was scored from 1 to 5, with 1 being unsatisfied and 5 being totally satisfied.

### Statistical analysis

The data processing and statistical analysis were conducted with MATLAB R2023a (The MathWorks Inc., Natick, United States). As there was no relevant prior information available, the sample size was determined not by calculating based on specific endpoints but rather by convenience sampling. The Lilliefors test was used for normality testing. For normally distributed data, results were expressed as mean ± standard deviation. A two-tailed paired *t*-test was used to compare the changes of the EIT parameters, FVC parameters, questionnaires, and other clinical parameters. Pearson’s linear correlation was used to compare the CAT score with FEV1 and FEV1/FVC (both global and regional). A *p*-value < 0.05 was considered statistically significant. Significance levels were corrected for multiple comparisons using Holm’s sequential Bonferroni method.

## Results

A total of 32 patients were included in the study. Eight patients were excluded due to the FVC maneuver not meeting the ATS criteria. Finally, data from 24 patients were analyzed (age, 59.5 ± 7.8 years old; height, 168.5 ± 6.4 cm; weight, 67.5 ± 10.6 kg). The FVC parameters and the questionnaires at the time of admission and discharge are presented in Table 1. Lung function was improved during hospitalization but was not significantly different. The symptoms of COPD were significantly improved. The differences in blood gases and other clinical parameters (PCT, CRP, WBC, IL-6, and LA) were not statistically significant.

**TABLE 1:** FVC parameters and questionnaires at admission and discharge of the study subjects.

	*FEV1* %pred		*FVC* %pred		*FEV1*/*FVC*		*PEF* %pred		mMRC		CAT	
	Adm	Dis	Adm	Dis	Adm	Dis	Adm	Dis	Adm	Dis	Adm	Dis
Mean	35.8	45.3	67.8	82.6	0.41	0.42	28.9	58.1	2.6	1.5	26.7	19.2
std	6.7	8.8	0.4	5.0	0.09	0.07	11.4	25.5	0.9	0.9	4.4	4.6
*p*-value	0.11		0.15		0.71		0.15		<0.001**		<0.001**	

Adm., admission; Dis., discharge; %pred., percent predicted; *FEV1*, forced expired volume at 1 s; *FVC*, forced vital capacity; *PEF*, peak expiratory flow; mMRC, Modified Medical Research Council; CAT, COPD assessment test scores. **p-value < 0.001.

**FIGURE 2:**
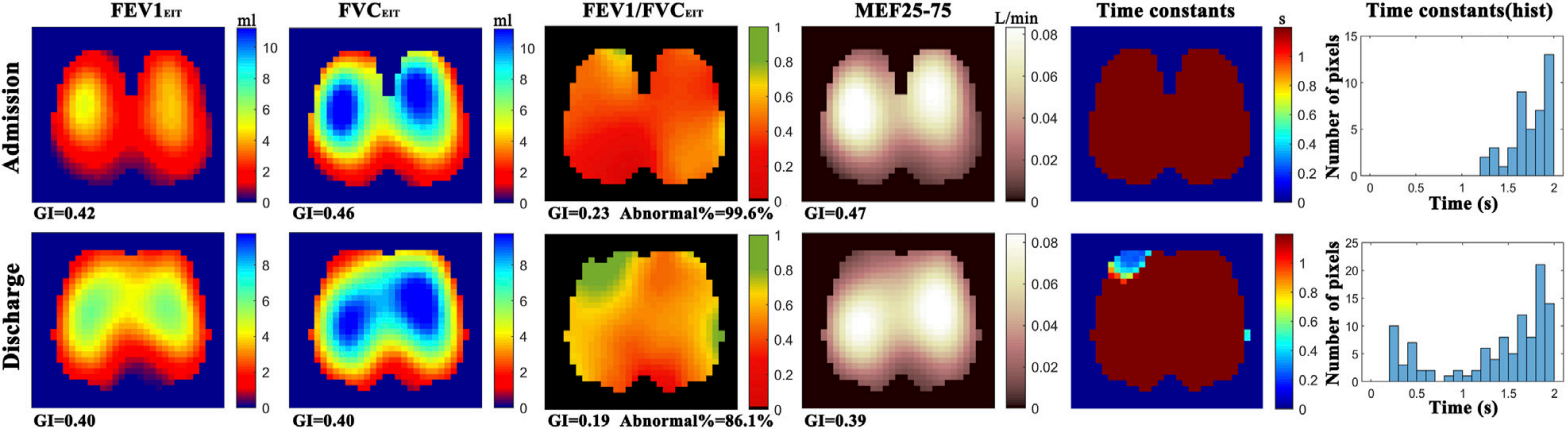
Functional EIT images showing the distribution of regional forced expiratory volume in 1s (*FEV1*), forced vital capacity (*FVC*), *FEV1/FVC*, mean expiratory flow between 25% and 75% of FVC (*MEF25-75*), and expiratory time constants. *GI*, global inhomogeneity. *Abnormal*%, percentage of the number of pixels with values < 0.7 over the total number of pixels in the lung regions. For the above-mentioned functional EIT images, each pixel corresponds to the functional parameter. Color bar on the right side of the subfigures indicates the level of the values of the corresponding parameters in each pixel. For example, in the functional image *FEV1/FVC*_*EIT*_, most of regions are red at admission (∼0.4), vs. green and yellow in some regions at discharge (∼0.7). Histogram of time constants (right column) showed the distribution of time constants of forced expiration. The average time is shorter, and the distribution is wider at discharge compared to that at admission.

**FIGURE 3:**
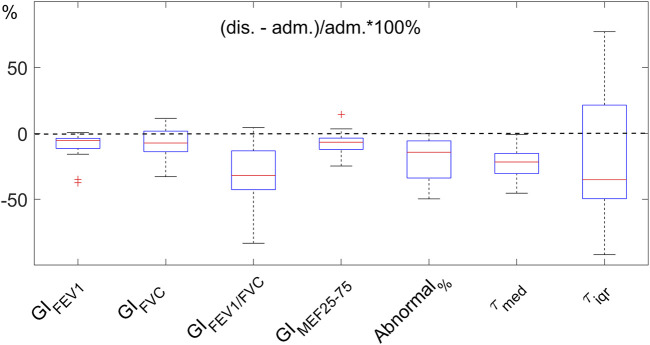
Improvement of EIT-based parameters after hospitalization. The parameters indicate the heterogeneity of the functional images (*GI*_*x*_, where x represents the parameters FEV1, FVC, FEV1/FVC, and MEF25-75), the regions with FEV1/FVC<0.7 (*Abnormal*%), and regional expiratory time constants (*τ*_*med*_ and *τ*_*iqr*_). The improvement is normalized to the values at admission (adm.). Dashed line indicates no change. The lower the values at discharge (dis.), the better. FEV1, forced expiratory volume in 1s. FVC, forced vital capacity. MEF25-75, mean expiratory flow between 25% and 75% of FVC. GI, global inhomogeneity. *Abnormal*%, percentage of the number of pixels with values < 0.7 over the total number of pixels in the lung regions.

**FIGURE 4:**
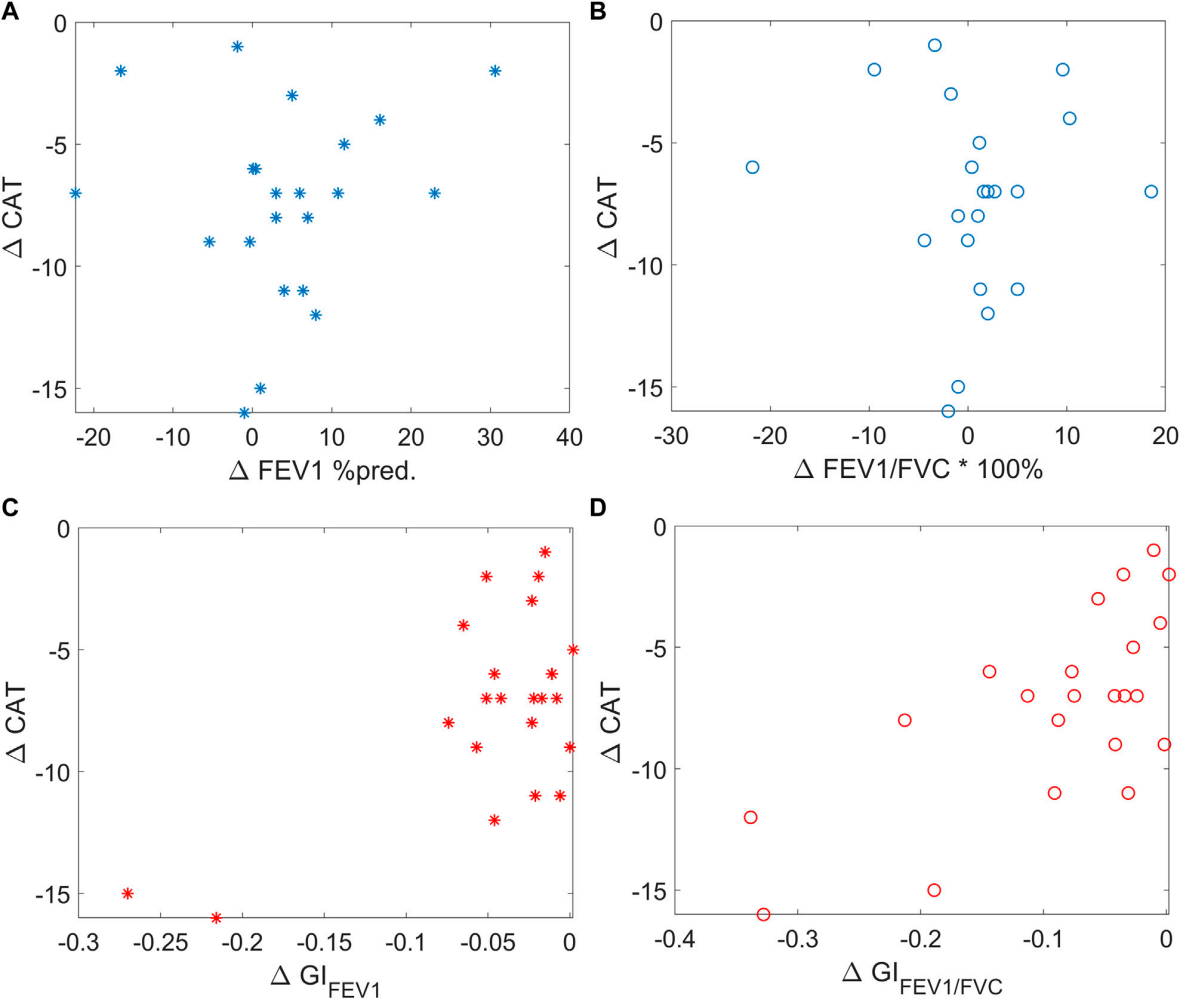
Scatter plots showing the correlation between ΔCAT and spirometry FEV1 (left top, blue stars), spirometry FEV1/FVC (right top, blue circles), EIT-based *GI*_*FEV1*_ (left bottom, red stars), and EIT-based *GI*_*FEV1/FVC*_ (right bottom, red circles). CAT, COPD assessment test; FEV1, forced expiratory volume in 1 s. FVC, forced vital capacity. MEF25-75, mean expiratory flow between 25% and 75% of FVC. *GI*, global inhomogeneity. *Abnormal*%, percentage of the number of pixels with values < 0.7 over the total number of pixels in the lung regions.

Example functional EIT images obtained from one of the studied patients are shown in Figure 2. These images present the spatial distribution of *FEV1*, *FVC*, *FEV1/FVC*, *MEF25-75*, and time constants. Each pixel in the images represents the corresponding functional parameters. It is observed that *FEV1*_*EIT*_, *FVC*_*EIT*_, and *FEV1/FVC*_*EIT*_ were improved at discharge (the pixel values were higher compared to that at admission). The time constants were distributed toward lower values (histogram). The values of EIT-based parameters are presented in Table 2. The improvement of the EIT-based parameters is presented in Figure 3 as the percentage to the values at the admission. All the parameters were significantly improved except τ_iqr_, which was improved but statistically insignificant. The correlations between the improvement of CAT and spirometry FEV1 and FEV1/FVC were low (R = 0.1 and −0.01, respectively). The correlations between ΔCAT and EIT-based heterogeneity (*GI*_*FEV1*_ and *GI*_*FEV1/FVC*_) were significant (R = 0.62 and 0.68, *p* = 0.002 and <0.001, respectively). The scatter plot in Figure 4 suggests that a high correlation between ΔCAT and Δ*GI*_*FEV1*_ was due to two outliers. However, ΔCAT and Δ*GI*_*FEV1/FVC*_ were highly correlated.

**TABLE 2:** EIT-based parameters at admission and discharge.

Parameter	Admission	Discharge	p
*GI*_*FEV1*_	0.46 ± 0.08	0.41 ± 0.03	0.002*
*GI*_*FVC*_	0.43 ± 0.05	0.40 ± 0.03	0.01*
*GI*_*FEV1/FVC*_	0.22 ± 0.12	0.13 ± 0.05	<0.001**
*GI*_*MEF25-75*_	0.45 ± 0.06	0.41 ± 0.03	<0.001**
*Abnormal*% (%)	82.2 ± 23.8	66.5 ± 26.1	<0.001**
*τ*_*med*_ (s)	1.53 ± 0.66	1.17 ± 0.53	<0.001**
*τ*_*iqr*_ (s)	0.70 ± 0.34	0.55 ± 0.31	0.06

*GI*_*x*_, global inhomogeneity index of the functional EIT, images; FEV1, forced expired volume at 1 s; FVC, forced vital capacity; MEF25-75, mean maximal expiratory flow between 25% and 75% vital capacity; *Abnormal*%, area of regions with FEV1/FVC<0.7; *τ*_*med*_, median regional time constant; *τ*_*iqr*_, interquartile range of the regional time constant. *p-value < 0.05; **p-value < 0.001.

The survey for EIT use satisfaction is summarized in Table 3. Six attending physicians and all 24 patients completed the survey. The overall acceptability of EIT measurement for patients was very high. The physicians were not familiar with the EIT procedure and considered the belt application a bit complicated. However, the measurement duration and the information provided by EIT were satisfactory.

**TABLE 3:** Surveys for the users’ satisfactory evaluation (translated) and the scores (ranging from 1 to 5, with 5 being most satisfied).

Doctor’s survey	Score	Patient’s survey	Score
Time required for applying EIT belt and conducting measurement	3.9 ± 1.4	Time required for applying EIT belt and conducting measurement	4.5 ± 0.5
EIT–FVC measurement process is easy	3.8 ± 0.8	EIT belt comfortability	4.9 ± 0.3
Duration to obtain the EIT measurement result	4.4 ± 0.5	EIT–FVC measurement process is easy and understandable	4.0 ± 0.4
Is the regional lung function information provided by EIT useful and helpful	4.0 ± 0.5	Is the EIT visual feedback and information providing useful information	4.8 ± 0.4
Overall satisfactory for EIT measurement	4.2 ± 0.9	Overall satisfactory for EIT measurement	4.0 ± 0.2

## Discussion

In the present study, we found that EIT captured the regional lung function improvement after COPD treatment. The results were highly correlated with patient-reported outcome assessments. Compared to the conventional spirometry FVC maneuver, EIT-based parameters were more sensitive. The overall acceptability of EIT measurement for both attending physicians and patients was high.

The COPD severity degree is defined by spirometry FVC parameters. Short hospitalization treatment of AECOPD improved the symptoms but did not significantly improve the spirometry parameters. For the diagnosis of AECOPD and criteria of admission, respiratory distress and significant symptoms not relieved by initial treatment are listed (NCBI, 2023). Ding et al. (2018) suggested that symptoms may be more related to physical activity impairment compared to lung function. We suspected that lung function reservoir is usually large, and global lung function parameters are usually impaired/improved with delay. In addition, unlike most diagnostic testing (e.g., blood tests and radiology), the FVC maneuver requires the patient to perform strenuous and precise physical procedures to capture accurate data. Although we have excluded eight patients with unacceptable spirometry data from the study, it was not possible to ensure that the patients had performed the maximum inhalation to total lung capacity or maximum expiratory efforts. The updates of ATS guidelines of 2019 implemented the “fourth phase” forced inspiration after the FVC as an extra measure to confirm the subject’s inspiratory effort (Graham et al., 2019). However, we cannot exclude the possibility that the insignificant differences in spirometry data between admission and discharge might be caused by the variation in the subjects’ inspiratory/expiratory efforts. On the other hand, regional lung function calculated with EIT data might be less dependent on the subjects’ effort. Frerichs et al. (2021) demonstrated that EIT-based ventilation inhomogeneity occurs not only during the FVC maneuver but also during normal tidal breathing. Therefore, pulmonary dysfunction or improvement might be identified without extreme cooperation of the COPD patients. In our present study, the FVC maneuver was scheduled for both admission and discharge. Therefore, simultaneous EIT measurement added no further burden to the patients. The accuracy and reliability of EIT have been compared previously with various standard modalities [e.g., (Frerichs et al., 2002; Hinz et al., 2003; Marquis et al., 2006)]. The EIT-derived flow should also be reliable as the volume changes are validated.

Both mMRC and CAT are recommended by the Global Initiative for Chronic Obstructive Lung Disease (GOLD) guidelines. Due to the items (symptoms) evaluated in two questionnaires, a discrepancy is expected between the two scoring systems (Rhee et al., 2015). Since the CAT scoring system better reflects the symptom severity (Cheng et al., 2019) and is less discrete compared to mMRC, we calculated the correlations between ΔCAT and spirometry and EIT-based parameters. The severity of COPD is defined with the FEV1 level and FEV1/FVC; hence, among the lung function parameters, we chose these two parameters. Patient-reported outcome assessments are widely accepted for clinical trials and drug development as important endpoints (Afroz et al., 2020). Nevertheless, such assessments are subjective. An objective measure is warranted to assess the treatment outcome. As demonstrated in the current study, blood tests and spirometry were not able to reflect the improvement for every individual. Previously, we demonstrated that EIT captured regional lung function information that was correlated with CT findings (Zhao et al., 2013). Theoretically, with a high temporal resolution and certain spatial resolution, EIT has much higher sensitivity than spirometry in terms of lung function and flow limitation. Shortening the treatment effect improving flow limitation was visualized with the EIT-based regional end-expiratory flow map (Zhao et al., 2020). In the present study, we demonstrated that the regional lung functions evaluated with EIT improved significantly (Table 2; Figure 1). Furthermore, EIT-based regional lung function was more sensitive and more related to symptom improvement (Figure 2). In addition to the conventional spirometry parameters, we also included the regional time constants into the analysis (Karagiannidis et al., 2018; Strodthoff et al., 2022). Time constant is a combination of resistance and compliance per definition (Karagiannidis et al., 2018; Strodthoff et al., 2022). In patients with COPD, its value significantly increases, and spatial heterogeneity is observed. As shown in the example in Figure 2, the time constant was high for most of the regions at admission, and after treatment, it was improved in some regions (Figure 2 last two columns on the right). The regional improvement during hospitalization enables the physician to judge the lung status from another angle besides flow limitation during the FVC maneuver. In the near future, we foresee that the EIT measurement can become a standard tool to assess the regional lung function. With such information, the physicians can better understand the lung status to plan individualized treatment programs and adjust the program according to the treatment effect on regional ventilation. With further development of the EIT technology, we hope that one day hardware would be as cheap as a peak flow meter so that all patients with COPD may have an EIT device at home to monitor their regional lung function and guide their daily respiratory exercises.

Although the ability of the EIT-based titration of positive end-expiratory pressure in ICU and perioperative has been validated (Pereira et al., 2018; Girrbach et al., 2020; He et al., 2021; Hsu et al., 2021), EIT is still a relatively novel imaging tool in respiratory medicine. A consensus statement of EIT examination and data analysis was published in 2017, where the measurement procedures were well-defined. However, for the attending physicians who learn to use EIT for the first time, the application of the electrode belt, patient management, etc. requires extra effort and time. Hence, the survey scores regarding these aspects were lower than those of other aspects (e.g., the value of the information; Table 3). The EIT imaging technology, on the other hand, is highly appreciated by the patients since the real-time visual feedback provided by EIT helps understand the ventilation status intuitively. In a previous study, EIT was used to guide chest physiotherapy, and the patients were also satisfied with the visual feedback, which increased the compliance of chest physiotherapy (Li et al., 2023). Therefore, we recommend that EIT be used as a bedside tool when developing patient-centered treatment programs.

We acknowledge several limitations to the present study. The number of participants was small, and due to the lack of prior information, the sample size was not calculated. No thresholds could be determined in terms of treatment effectiveness. Nevertheless, our data demonstrate the improvement of regional lung function heterogeneity within 1 week of hospitalization and serve as prior knowledge for further study development. The limitations to EIT technology are noted as well. Electrode placement (Frerichs et al., 2024) or patient positioning (Yang et al., 2023b) has an influence on the EIT results. Therefore, to minimize the potential sources of bias in EIT measurements, EIT results should be compared only with the same measurement plane and body position. In addition, EIT cannot render the absolute volume, so only the distribution and the relative impedance change and not the absolute ones between two time points are comparable.

## Conclusion

EIT-derived regional lung function was highly correlated with the patient-reported outcome assessment. It was feasible to use EIT as an objective bedside evaluation tool for COPD treatment efficacy. The sensitivity of regional lung function provided by EIT was higher than the spirometry global parameters in respect to symptom improvement. Future studies may focus on the impact of EIT monitoring on the patients’ clinical outcomes.

## Data Availability

The original contributions presented in the study are included in the article/Supplementary material; further inquiries can be directed to the corresponding authors.
